# FDG-PET Lacks Sufficient Sensitivity to Detect Myxoid Liposarcoma Spinal Metastases Detected by MRI

**DOI:** 10.1155/2007/36785

**Published:** 2007-05-23

**Authors:** Joseph H. Schwab, John H. Healey

**Affiliations:** Section of Orthopedic Surgery, Department of Surgery, Memorial Sloan Kettering Cancer Center, Weill Cornell University Medical School, New York, NY 10021, USA

## Abstract

*Purpose*. To document a case of myxoid liposarcoma in which PET scan was less sensitive than MRI in detecting spinal metastasis. *Materials and Methods*. The case of a 65-year-old female with a history of myxoid liposarcoma (MLS) of the thigh resected 5 years previously and now presenting with low back pain is presented. Her medical oncologist ordered an FDG-PET scan to evaluate distant recurrence. Subsequently, an MRI of her spine was obtained by her surgeon. *Results*. The FDG-PET scan was obtained 1 week prior to the MRI, and it did not show increased glucose uptake in the spine. Her MRI did show increased signal intensity in her lumbar spine. CT needle biopsy confirmed the lesion to be metastatic MLS. *Conclusion*. FDG-PET scans are utilized to detect distant recurrence of cancerous lesions. Myxoid liposarcoma has a unique propensity to metastasize to the spine. Previous reports have documented the unreliability of bone scintigraphy to diagnose these metastases. Our report demonstrates that FDG-PET may also lack the sensitivity needed to detect these lesions. We advocate total spine MRI when screening for metastases in this population when they present with back pain.

Liposarcoma is one of the most common soft tissue sarcomas and myxoid liposarcoma (MLS) is the second most common subtype. Soft tissue sarcomas tend to metastasize to the lungs, however myxoid liposarcoma may metastasize to unusual extrapulmonary sites including bone [1–3]. Several reports have documented cases of metastatic MLS to the spine. It is unclear which imaging modality has the greatest ability to detect these lesions. Bone scintigraphy has been shown to be unreliable [4–6]. FDG-PET scans are being utilized in many centers to detect metastases, however their sensitivity and specificity is not known. We present a case of metastatic MLS to the spine which was not detected by FDG-PET but had a positive MRI.

## 1. CASE REPORT

**Figure 1:**
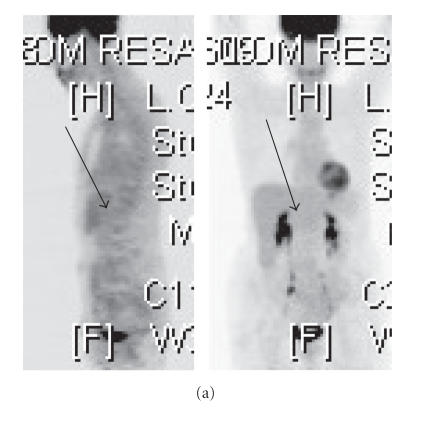

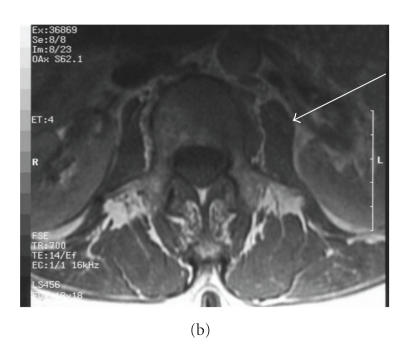

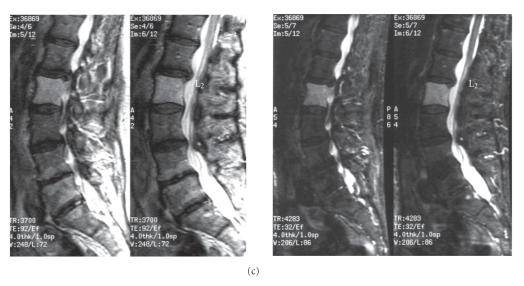
(a) The PET scan did not demonstrate increased glucose uptake in the lumbar spine. (b) and (c) Axial and sagittal MRI demonsrated abnormal signal intensity in the second lumber vertebrae.

A 65-year-old female with a history of an AJCC stage III popliteal myxoid liposarcoma developed mild low back pain 5 years after her initial surgery. The primary tumor had been treated with wide resection followed by 6500 cGy to the popliteal fossa. She had been disease-free and was seen by her medical oncologist for a routine check when she complained of back pain. Whole body FDG-PET (Figure 1(a)) did not show increased glucose uptake in the lumbar spine (*black arrow*). Subsequent axial MRI (Figure 1(b)) showed a soft tissue component to a mass in the lumbar spine (*white arrow*). Sagittal MRI (Figure 1(c)) of the lumbar spine demonstrates a lesion in the second lumbar vertebrae on T1 and T2 weighted images. A CT-guided biopsy confirmed the diagnosis of metastatic myxoid liposarcoma. The patient was treated with intensity modulated radiation therapy (IMRT). Her back pain subsided but she developed further systemic disease and died.

## 2. DISCUSSION

Screening for metastatic liposarcoma is controversial. Chest CT misses a significant number of metastases as MLS has a propensity to metastasize to extrapulmonary sites such as the one in our case report. Bone scintigraphy is not a reliable diagnostic test for metastatic myxoid liposarcoma [4–6]. This case demonstrates that FDG-PET scanning may lack sufficient sensitivity in diagnosing MLS metastases. The low degree of glucose uptake may reflect low metabolic activity found in the myxoid/paucicelluar regions of these tumors. MRI appears to be the most reliable method of diagnosing spinal metastasis in myxoid liposarcoma.

The negative PET scan may reflect low metabolic activity within the myxoid/paucicelluar regions of these tumors. It is possible that the absence of appreciable glucose uptake reflects the inability of the PET scans to detect those cells that are actively utilizing glucose within the myxoid stroma. The radiolabeled glucose may not be able to reach the cells embedded in the matrix. However, our case represents a high-grade liposarcoma with at least a 5% round cell component. One would expect that these areas could be detected by FDG-PET. Further study is necessary to determine whether the ratio of round cells relative to myxoid stroma is important.

Spine metastasis should be considered when one is evaluating a patient with a history of MLS presenting with back pain. Bone scintigraphy and FDG-PET do not seem to be sensitive enough to detect these lesions. We recommend MRI of the spine when one is confronted with a patient complaining of back pain with a history of myxoid liposarcoma. While MRI seems to be more sensitive than FDG-PET, neither is specific, and biopsy should be considered for histologic confirmation.
